# Editorial: Pathogenesis of diabetic foot ulcers

**DOI:** 10.3389/fphar.2023.1218201

**Published:** 2023-05-19

**Authors:** Zhihong Peng, Jean-Philippe Lavigne

**Affiliations:** ^1^ National and Local Joint Engineering Research Center of High-Throughput Drug Screening Technology, State Key Laboratory of Biocatalysis and Enzyme Engineering, College of Health Science and Engineering, Hubei University, Wuhan, China; ^2^ Department of Microbiology and Hospital Hygiene, VBIC, INSERM 1047, University Montpellier, CHU Nîmes, Nîmes, France

**Keywords:** diabetic foot ulcers, pathogenesis, treatment, wound healing, chronic wound

Diabetes mellitus is a complex metabolic disease that affects more than 340 million individuals worldwide. Around 20%–25% of these patients develop diabetic wounds mainly localized on the foot during their life (Edmonds et al., 2021). Diabetic foot ulcers (DFUs) are a common complication of diabetes mellitus due to the triopathy associating ischemia, peripheral neuropathy and arteriopathy. Morbidity and mortality of DFUs depend on the studied population and other factors, such as access to healthcare, diabetes management, and comorbidities. According to the World Health Organization (WHO), the global prevalence and incidence of DFUs are approximately 6.3% and 19.4%, respectively (Paisey et al., 2019). In addition, DFUs are the primary cause of non-traumatic lower extremity amputations, accounting for up to 70% of non-traumatic lower extremity amputations. Although becaplermin is the only FDA-approved medication for treating DFUs, it is not typically the first-line therapy due to its low efficacy and increased risk of cancer and mortality (Ziyadeh et al., 2011). The absence of effective and safe treatment has necessitated the development of more effective medications or procedures. The development of DFU is a serious complication for patients living with diabetes mellitus. Their mechanism of the development remains partially unknown. A better understanding of pathogenic variables can aid in the development of innovative treatments for poor healing. Therefore, this Research Topic aims to provide a forum focusing on the pathogenesis of DFUs and the innovative therapies and drugs for this pathology.

During DFUs, the process of wound healing is hindered by several mechanisms. These include low growth factor activity, reduced cellular proliferation, elevated inflammatory marker, poor neovascularization, and imbalance in extracellular matrix (ECM) synthesis and degradation. The matrix metalloproteinases (MMPs) play a crucial role in all these processes (Chang and Nguyen, 2021). In the review article, Fu et al. extensively discussed the significance of MMPs in wound healing and analyzed the differences in MMPs expression between normal and chronic wounds. The review concluded that the overexpression of MMPs is a characteristic feature of DFUs, and the elevated levels of MMPs in DFUs lead to tissue degradation and impaired wound healing. Precise regulation of MMP levels in DFUs could promote faster and substantial wound healing. Moreover, the authors also highlighted that MMP-9 is particularly overexpressed in DFUs and could be a promising target for treating DFUs.

Infection is a common occurrence in DFUs, and inhibiting bacterial growth is crucial for promoting the wound healing (Peng et al., 2021). Nano-silver is a particle processed using nanotechnology, which grants it strong skin permeability and excellent antibacterial effect. Epidermal growth factor (EGF) has gained popularity in wound treatment due to its ability to encourage epidermal cell proliferation and rebuild blood vessels. In a study conducted by Zhang et al., it was found that the combination of nano-silver dressing with EGF for treating DFUs is highly effective in inhibiting bacterial proliferation, controlling wound infection, accelerating epidermal growth and granulation tissue formation, and ultimately promoting wound healing. This innovative approach provides valuable insights into the management of DFUs.

Stem cells possess the ability to induce differentiation and release cytokines, which can help to form a capillary network in ischemic tissues. This process can establish collateral circulation, enhance blood perfusion, and improve the ischemic condition of affected tissues, resulting in a therapeutic outcome for patients (Krasilnikova et al., 2022). Li et al.’s research, which utilized scRNA-seq, characterized the microenvironment of DFUs and identified potential drugs. The authors discovered that three genes (CD19, ITGAM, and HLA-DR) were expressed at higher levels in DFUs. Based on the Drug Gene Network’s findings, cyclosporine, simvastatin, curcumin, luteolin, apigenin, and chrysin are all promising drugs for treating DFUs, making them valuable resources for DFU treatment.

Foot ulceration is a preventable ailment and modest interventions can reduce amputations and death by as much as 70% by implementing risk factor lowering programs. Identifying the risk factors associated with this ailment can help healthcare providers develop more effective prevention methods, benefitting the patients’ quality of life. Wang et al. evaluated the morbidity and mortality risk factors of DFUs and found that age and albumin levels were independent predictors of mortality. Additionally, by investigating the effects of negative pressure wound therapy (NPWT) combined with platelet-rich plasma-fibrin glue (PRP) on DFUs, the authors observed that this treatment accelerated wound healing and reduced mortality rate, providing valuable information for the management of this condition.

Our aspiration is that the reader will discover a valuable reference in this Research Topic, showcasing the current state of the art in understanding the mechanisms of DFUs, and identifying potential solutions to enhance wound healing. We firmly believe that this will offer future strategies for managing DFUs effectively.

## References

[B1] Chang M. Nguyen T. T. (2021). Strategy for treatment of infected diabetic foot ulcers. Accounts Chem. Res. 54 (5), 1080–1093. 10.1021/acs.accounts.0c00864 33596041

[B2] Edmonds M. Manu C. Vas P. (2021). The current burden of diabetic foot disease. J. Clin. Orthop. trauma 17, 88–93. 10.1016/j.jcot.2021.01.017 33680841 PMC7919962

[B3] Krasilnikova O. Baranovskii D. Lyundup A. Shegay P. Kaprin A. Klabukov I. (2022). Stem and somatic cell monotherapy for the treatment of diabetic foot ulcers: Review of clinical studies and mechanisms of action. Stem Cell Rev. Rep. 18 (6), 1974–1985. 10.1007/s12015-022-10379-z 35476187

[B4] Paisey R. B. Abbott A. Paisey C. F. Walker D. (2019). Diabetic foot ulcer incidence and survival with improved diabetic foot services: An 18‐year study. Diabet. Med. 36 (11), 1424–1430. 10.1111/dme.14045 31150130 PMC6852104

[B5] Peng Z. Nguyen T. T. Song W. Anderson B. Wolter W. R. Schroeder V. A. (2021). Selective MMP-9 inhibitor (R)-ND-336 alone or in combination with linezolid accelerates wound healing in infected diabetic mice. ACS Pharmacol. Transl. Sci. 4 (1), 107–117. 10.1021/acsptsci.0c00104 33615165 PMC7887744

[B6] Ziyadeh N. Fife D. Walker A. M. Wilkinson G. S. Seeger J. D. (2011). A matched cohort study of the risk of cancer in users of becaplermin. Adv. skin wound care 24 (1), 31–39. 10.1097/01.asw.0000392922.30229.b3 21173589

